# Sedentary Behaviors and Health Outcomes among Young Adults: A Systematic Review of Longitudinal Studies

**DOI:** 10.3390/healthcare10081480

**Published:** 2022-08-06

**Authors:** Zan Huang, Yanjie Liu, Yulan Zhou

**Affiliations:** College of Physical Education and Health Sciences, Zhejiang Normal University, Jinhua 321004, China

**Keywords:** health, sedentary behavior, young adults, longitudinal studies

## Abstract

Objective: This study aimed to review and provide an informative synthesis of the findings from longitudinal studies that describe the relationship between sedentary behavior and various health outcomes among young adults. Methods: A literature search was conducted in Web of Science, PubMed, APA PsycInfo, MEDLINE, Embase, and the Cochrane Library for articles that examined the association between sedentary behavior and health outcomes among young adults aged 18–34 years. Two reviewers independently examined the articles and performed data extraction and quality assessment. The level of evidence was determined using the best-evidence synthesis. Results: A total of 34 studies were included in the analysis, 18 of which were high-quality studies. On the basis of inconsistency in the findings among studies, insufficient evidence was concluded for sedentary behavior and adiposity indicators, physical fitness, metabolic syndrome/cardiovascular disease risk factors, cognitive function, and mood disorders. Based on one high-quality study, moderate evidence for a negative relationship between sedentary behavior and physical fitness was observed. Conclusions: Given the trend toward increased time in sedentary behaviors and the inconsistent current findings, additional longitudinal studies of high methodologic quality are recommended to clarify the relationships between sedentary behavior and health outcomes among young adults.

## 1. Introduction

Sedentary behavior, an important area of study in health research, is defined as activities that involve energy expenditure ≤1.5 metabolic equivalent units such as sleeping, sitting, lying down, watching TV, and other forms of screen-based entertainment [1]. Given the increasing availability of information and communication technology and labor-saving devices, people currently spend a lot of time on sedentary behaviors around the world [2,3]. By using data from a representative sample of the National Health and Nutrition Examination Surveys, Du et al., 2019 found that adults in the United States of America spending time on sedentary behaviors increased from 5.7 h per day in 2007–2008 to 6.4 h per day in 2015–2016 [4]. Australian adults sit an average of 8.8 h per day [5]. In addition, a study of four European countries (i.e., the United Kingdom, Portugal, Norway, and Sweden) showed that adults were sedentary for 8.8 h per day, as measured by accelerometers [6]. Sedentary behavior and physical activity are two distinct behaviors and are likely to have independent effects on health indicators [7]. Many adults who achieve the 60 min moderate-to-vigorous physical activity recommended by the World Health Organization may still be at increased risk of ill health effect due to prolonged engagement in sedentary behaviors for the rest of the day [8].

To strengthen the evidence based on sedentary behavior as an adult health risk, a large number of longitudinal studies have been conducted to investigate the association between sedentary behavior and health outcomes among adults, but the findings have been mixed [9,10,11,12,13]. Some studies have reported that sedentary behavior is associated with health outcomes. For example, Vaara et al., 2020 identified that total sedentary time could increase the body mass index, waist circumference, and body fat of adults (mean age 28.5 years) [9]. Hoang et al., 2016 showed that prolonged television viewing led to decreased cognitive function for adults aged 18–30 years [10], and Thomée et al., 2007 found that computer use was positively associated with mood disorders among 20–24 year old young adults [11]. However, some studies have reported no association. Staiano et al., 2018 found no correlation between sedentary time and obesity in adults aged 20 to 35 [12]. Carter et al., 2020 reported no association between the amount of time that adults (mean age 33.6 years) spent sitting in the workplace and cognitive function [13]. Inconsistent results have indicated that existing studies should be systematically summarized and analyzed to have an improved understanding of the association between sedentary and health outcomes.

To date, two systematic reviews have focused on the longitudinal studies that examined the relationship between sedentary behavior and health outcomes among adults (range = 18–90 years) [14,15]. Nevertheless, remarkable differences exist in the physical and psychological characteristics of different age groups, which cannot be extended to the current findings to the whole of adulthood [16,17]. The relationship between sedentary and health outcomes in specific age groups should be systematically reviewed. Young adulthood (18–34 years) is a critical life period during which young people leave home, begin university, enter the workforce, and establish a family [18]. Changes at this stage affect the physical health of young adults, which is the foundation of their health in old age [19]. In addition, adults during this period may be susceptible to mental health problems such as depression and anxiety, and suicide [20]. It is necessary to explore the impact of sedentary behaviors on the health outcomes among young adults to provide references for the further research and implementation of subsequent interventions. Therefore, this systematic review aimed to evaluate and synthesize the longitudinal research on the relationship between sedentary behavior and health outcomes in young adults as well as analyze the evidence that sedentary behavior leads to health risks in young adults.

## 2. Materials and Methods

### 2.1. Literature Search

A systematic search of the relevant studies in Web of Science, PubMed, APA PsycInfo, MEDLINE, Embase, and the Cochrane Library was conducted from the inception of the databases up to May 2022. The search strategy included a combination of three main areas: (1) sedentary behavior: sedentary behavior OR physical inactivity OR sedentary OR sedentary lifestyle OR sitting OR television OR computer OR TV OR screen time OR video game OR social media OR internet; (2) health outcomes: adiposity indicators (overweight OR obesity OR body mass index OR waist circumference OR skinfolds), physical fitness (bone mineral density OR muscle strength OR muscle endurance OR cardiorespiratory), metabolic syndrome/cardiovascular disease risk factors (metabolic syndrome OR hypertension OR diabete* OR cardiovascular disease*), cognitive function (attention OR memor*), and emotional disorder (depress* OR anxiety OR stress); and (3) young adult: young adult* or adult* or college student or postgraduate*. The complete search terms and search strategies used in each database are shown in the Supplementary Materials. In addition, a manual search was conducted for all reference lists to determine additional relevant papers.

### 2.2. Inclusion and Exclusion Criteria

The inclusion and exclusion criteria for this review were as follows. (1) Peer-reviewed journals with full-text studies published in the English language until May 2022 were included. Unpublished articles, conference papers, doctoral dissertations, monographs, and literature reviews were excluded. (2) Studies that presented longitudinal data on the relationship between sedentary behavior and health outcomes among young adults were included. Cross-sectional and intervention research (i.e., implementing a change to the usual condition in order to increase or reduce sedentary behavior) were excluded. Longitudinal studies vary enormously in the duration of follow-up (from a few days or weeks to over decades) [21]. No limitations were set regarding the follow-up period. (3) Studies with samples of healthy population (i.e., no psychiatric, mental, or physical illness) aged 18–34 years were included, and other age groups and specific patient groups or samples identified by diseases were excluded. (4) When multiple studies conducted analyses on the same sample, the study with the largest sample size and most comprehensive information was included, and other studies were excluded.

### 2.3. Data Extraction and Quality Assessment

Two researchers independently performed the data extraction and quality assessment of all of the included articles and discussed the inconsistent results until an agreement was reached [22]. Data including first author, published year, country, study population (sample size, age, and gender), follow-up duration, type and measurement of sedentary behavior, type and measurement of health indicators, confounding factors, statistical analysis, and the main results were extracted from all of the included studies.

The quality of the included articles was evaluated using criteria adapted from the Quality of Reporting of Observational Longitudinal Research and the Evaluation of the Quality of Prognosis Studies in Systematic Reviews [23,24]. The quality assessment criteria consisted of 15 items and comprehensively evaluated the quality of articles from four dimensions: (1) study population and participation (four items); (2) study attrition (four items), (3) data collection (three items), and (4) data analysis (four items). The reviewers independently rated each criterion as “+” (positive, thoroughly and clearly described), “−“ (negative, not illustrated), or “?” (insufficiently described) on the basis of the information provided in the articles. All disagreements were discussed until an agreement was reached. A study was considered to be of high quality if more than 50% of the methodological criteria was scored positively. Otherwise, the study was considered to be of low quality [25].

### 2.4. Evidence Synthesis

After summarizing the included studies, the results showed that studies were heterogeneous in terms of statistical analyses, type and measurement of sedentary behavior, and health outcome. A formal meta-analysis is thought to be inappropriate. Therefore, the best-evidence synthesis was applied to synthesize the methodological quality of the studies and provide conclusions on the association between sedentary behavior and health outcomes [25,26]. This method consisted of the following levels: (1) strong evidence: consistent findings in multiple high-quality studies (≥2); (2) moderate evidence: consistent findings in one high-quality study and at least one low-quality study or multiple low-quality studies with consistent findings; and (3) insufficient evidence: only one available study or inconsistent findings in multiple studies (≥2) [25,26]. Similar to previous reviews that used this best-evidence synthesis, consistency was defined on two levels: (1) within a study (i.e., ≥75% of results in same direction within a study) to account for multiple results in the same health indicator category and (2) between studies (i.e., ≥75% of results in same direction across studies examined) [27]. If one study contained two or more different categories of health outcomes, each category was analyzed separately.

## 3. Results

### 3.1. Study Selection

**Figure 1:**
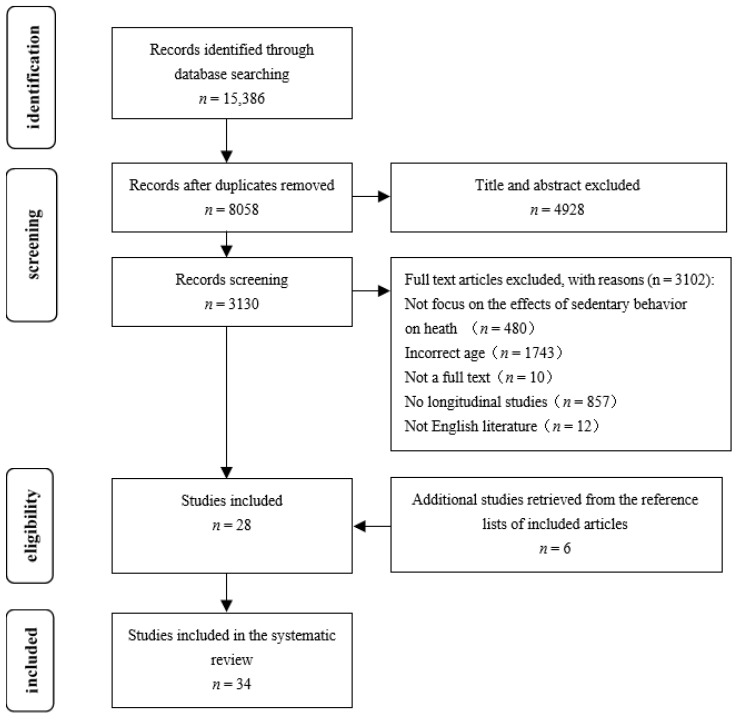
The flow diagram of the study selection process.

The search resulted in 15,386 studies (3385 from Web of Science, 3130 from PubMed, 1055 from APA PsycInfo, 2706 from MEDLINE, 3521 from Embase, and 1589 from the Cochrane Library). After removing duplicate publications, 8058 publications remained. After screening the titles and abstracts, 3130 studies were identified as potentially relevant, and full-texts were then obtained. Afterward, 28 studies met the inclusion and exclusion criteria. The search of reference lists from relevant papers and reviews yielded six more publications. Therefore, 34 studies were included in this review (Figure 1). The inter-rater agreement for all data extracted from the included studies was 85.2%. We resolved any disagreements by discussion.

### 3.2. Characteristics of the Included Studies

Table 1 shows the characteristics of the included articles in this review. In the included literature published from 1998 to 2022, two, seven, and 25 studies were published before 2000, from 2001 to 2010, and from 2011 to 2022, respectively. Investigations were primarily conducted in the United States (10 studies) followed by the United Kingdom (eight studies) and Australia (five studies); New Zealand, the Netherlands, Brazil, and Switzerland had two articles each; Finland, Japan, and Spain had one study each. The total sample size of the literature was 10,772 participants, with 15 studies having less than 1000 participants and 19 studies having over 1000 participants. Among the reviewed literature, 12, eight, and 10 studies reported the mean ages of 18–25, 25–30, and 30–34 years, respectively. The four remaining studies were birth cohort studies.

A total of five, 17, and 12 studies reported follow-up periods of <1, 1–5, >5 years, respectively. Most studies (23 studies) used subjective methods to assess sedentary behavior (e.g., self-reported questionnaires), and the 11 remaining studies used objective methods (e.g., accelerometers). A total of 19 studies compositely measured the effect of all sedentary behaviors on health outcomes, and 15 studies examined the influences of specific types of sedentary behavior (e.g., computer use, TV viewing, and cellphone use) on the health outcomes. Most studies (20 studies) controlled the influence of age, gender, race, and education level on the results, and a few studies (14 studies) controlled the effect of physical activity and sleep. A total of 33 studies used multivariate analysis methods (e.g., linear regression analysis, logistic regression analysis, and generalized estimating equations) to examine the relationship between sedentary behavior and health outcomes. Only one article used univariate analysis methods (e.g., ANOVA).

### 3.3. Methodological Quality

The initial agreement in the methodological quality assessment was 82.2% between the two reviewers. Table 2 provides the methodological quality assessment per study. For the study population and participation, among the 34 studies, 26 clearly described the source population, three reported the sampling frame and recruitment methods, 12 had a participation rate at baseline of at least 80%, and 23 described the baseline sample. For the study attrition, among the 34 studies, 14 reported the exact number at each follow-up measurement, 34 clearly described follow-up duration, eight had a sufficient response rate at follow-up, and six reported that the nonresponse at follow-up was not selective. For data collection, 11 studies used objective measurement for sedentary behavior, 26 studies assessed sedentary behavior at a time prior to the measurement of the health outcome, and four studies used objective measurement for the health outcome. For data analysis, 33 studies used an appropriate statistical model, 18 studies had at least 10 times the number of the independent variables, 32 studies described the point estimates and measures of variability, and 11 studies did not perform the selective reporting of results. The quality scores of the included studies ranged from 20% to 73%. A total of 18 out of the 34 studies scored higher than 50% and were categorized as high quality, and 16 studies were categorized as low quality.

### 3.4. Summary of Results

Out of the 34 studies included in this review, three studies reported two different categories of health outcomes [9,13,53]. Therefore, 37 studies (representing 34 articles) examined the association between sedentary behavior and health outcomes among young adults, and 12 studies reported data on adiposity indicators. A total of two, 11, two, and 10 studies reported the physical fitness, metabolic syndrome/cardiovascular disease risk factors, cognitive function, and emotional disorder, respectively (Table 3).

#### 3.4.1. Sedentary Behavior: Adiposity Indicators

Twelve studies examined the relationship between sedentary behavior and adiposity indicators (e.g., waist circumference, body fat percentage, and BMI). Five studies examined the sedentary time in relation to body fat mass such as waist circumference, body fat percentage, or skinfold thickness. Of these five studies, two studies found that the sedentary time was associated with body fat percentage and waist and hip circumference gain [9,54], whereas three studies showed no association between sedentary behavior and body fat percentage [33], waist circumference [37], and visceral abdominal fat [12]. Seven studies examined the relationship between sedentary behavior and BMI. Of these seven studies, five found that sedentary behavior could increase the BMI [47,48,50,51,53], and two showed no relationship between sedentary behavior and BMI [35,55]. Out of 12 studies, seven (58.3% including one high-quality study [9]) reported a positive relationship between sedentary behavior and adiposity indicators. Based on the inconsistent findings among the studies identified, insufficient evidence for the relationship between sedentary behavior and adiposity gain was observed.

#### 3.4.2. Sedentary Behavior: Physical Fitness

Two studies examined the relationship between sedentary behavior and physical fitness (e.g., cardiorespiratory and muscle fitness) and indicated a negative association between sedentary behavior and cardiorespiratory fitness [9,53]. In addition, Vaara et al., 2020 examined the relationship between sedentary behavior and muscle fitness and found a negative association [9]. Two studies (100% including one high-quality study [9]) examined the negative effects of sedentary behavior on cardiorespiratory and muscle fitness. Based on the best-evidence synthesis, moderate evidence for a negative association between sedentary behavior and physical fitness was observed.

#### 3.4.3. Sedentary Behavior: Metabolic Syndrome/Cardiovascular Disease Risk Factors

Eleven studies investigated the relationship between sedentary behavior and metabolic syndrome/cardiovascular disease risk factors (e.g., triglycerides, HDL cholesterol, glucose, insulin, and blood pressure). Two studies showed that sedentary behavior was positively associated with triglycerides and negatively associated with HDL cholesterol [28,29]. Hancox et al., 2004 identified that sedentary behavior was a predictor of an increase in total serum cholesterol [49]. Whitaker et al., 2019 showed that prolonged sedentary behavior may increase the composite risk of cardiovascular disease [36]. Fujii et al., 2021 investigated the clinical effect of sedentary behavior on chronic kidney disease and showed that sedentary behavior was a significant predictor of the incidence of proteinuria in men [42]. Of the remaining six studies, three showed that sedentary behavior was not associated with HDL, LDL, triglycerides, and serum insulin [30,32,43]. Two studies showed no association between different sedentary behaviors (e.g., television viewing, computer use, and driving) and self-reported prevalence of hypertension [31,52]. In addition, Stamatakis et al., 2012 investigated the association between sedentary behavior and cardiometabolic risk and found no association [38]. Out of 11 studies, five (45.4% including three high-quality studies [28,36,42]) reported a remarkable effect of sedentary behavior on the metabolic syndrome/cardiovascular disease risk factors. Based on the inconsistent findings among the studies, the evidence to indicate a substantial association between sedentary behavior and the risk of metabolic syndrome/cardiovascular disease is insufficient.

#### 3.4.4. Sedentary Behavior: Cognitive Function

Two studies investigated the correlation between sedentary behavior and cognitive function. Hoang et al., 2016 found that compared with adults with low sedentary behavior time, those with high sedentary behavior time had low levels of processing speed and executive function [10]. However, Carter et al., 2020 reported no association between sedentary behavior and executive function, working memory, and attention [13]. Of the two studies, only one high-quality study [10] (50%) showed a negative association between sedentary behavior and cognitive function, indicating that we found insufficient evidence for a relationship between sedentary behavior and a decline in cognitive function.

#### 3.4.5. Sedentary Behavior: Emotional Disorder

Ten studies examined the relationship between sedentary behavior and emotional disorders (e.g., stress, sleep disturbance, depression, anxiety, and anger). Two studies found a positive association between sedentary behavior and depression [39,56]. DeMello et al., 2018 identified a positive association between sedentary behavior and total mood disturbance [34]. Another study indicated that increases in sedentary time were associated with high mood disturbance and stress [40]. Of the remaining six studies, one examined the association between sedentary behavior and psychological distress (e.g., sadness, tension, irritability, despair, powerlessness, and worthlessness) and found no association [41]. Carter et al., 2020 found no relationship between sedentary behavior and mood [13]. Four other studies showed no correlation between sedentary behavior and anxiety, stress, and depression [11,44,45,46]. Of the 10 studies, four (40% including two high-quality studies [34,40]) indicated that sedentary behavior resulted in emotional disorders. According to the best-evidence synthesis, the evidence for the positive association between sedentary behavior and emotional disorders is insufficient.

## 4. Discussion

The present review aimed to systematically summarize the literature with regard to the longitudinal relationship between sedentary behavior and health outcomes among young adults, taking into account the methodological quality of the studies. Despite the start date of the literature being 1998, the majority (25 of 34 studies) of the studies included were published after 2010. This indicates that over recent years, the topic of sedentary behavior as an independent predictor for health outcomes has gained much attention in the literature. Based on the studies identified, moderate evidence for the negative relationship between sedentary behavior and physical fitness was found. Furthermore, insufficient evidence was found for the relationship between sedentary behavior and adiposity indicators, metabolic syndrome/cardiovascular disease risk factors, cognitive function, and emotional disorder.

The insufficient evidence for the longitudinal relationship between sedentary behavior and adiposity indicators supports the earlier systematic review of Proper et al., 2011 [14]. This result may be due to the different adiposity indicators of the included studies. In this review, adiposity indicators included body fat mass (waist circumference, body fat percentage, hip circumference, skinfold thickness, and WHR) and BMI/body weight. Body fat percentage is the percentage of body fat in body weight, which distinguish whether the increase in body weight is caused by muscle or fat increase [57]. However, BMI evaluates the degree of obesity by the ratio of weight to height that reflect the body’s fat and muscle content to a certain extent [58]. Previous cross-sectional evidence has suggested that the time spent in sedentary behavior leads to the increase in adiposity, especially the accumulation of abdominal fat [59]. The effect of sedentary behavior on BMI did not distinguish the degree of the influence of fat and muscle mass, resulting in the inconsistent results of the current review. In addition, the adiposity indicators were measured in a variety of ways including tape measure, electronic scales, X-ray and ultrasound scanners, etc. The longitudinal studies conducted by Staiano et al., 2018 and Silva et al., 2019 found that there was no significant relationship between sedentary behavior and adiposity indicators when it was measured by X-ray and ultrasound scanners [12,37]. Conversely, van de Laar et al., 2014 and Vieira et al., 2020 identified a significant longitudinal association between sedentary behavior and adiposity indicators, which was measured using simple tools (tape measure, electronic scales) [29,43]. The findings imply that future studies need to distinguish the different dimensions of adiposity indicators and use standardized measures to assess the relationship between sedentary behavior and adiposity indicators.

Two studies found a significant negative association between sedentary behavior and physical fitness. The moderate evidence for the association was obtained since only one high-quality study was available. Long-term sedentary behavior could cause venous blood pooling in the lower limbs, which could decrease muscle blood flow and inadequate muscle contraction [60]. More time spent in sedentary behavior can also cause impairment in forced vital capacity and forced vital capacity in 1 s, and directly leading to decreased muscle and cardiorespiratory fitness [61]. Moreover, the positive influence of five weeks of physical activity intervention on cardiorespiratory and muscle fitness has been well-documented [62]. Adults that spend more time in sedentary behavior tend to spend less time being physically active than those with shorter sedentary behavior time, which indirectly leads to a decline in cardiorespiratory and muscle fitness [63]. However, this result should be interpreted cautiously, given that it was only based on one high-quality study.

There have been inconsistences in the studies on sedentary behavior and metabolic syndrome/cardiovascular disease risk factors (45.4%), leading to insufficient evidence. The result was consistent with previous reviews [14,15]. Two main reasons could explain the inconsistent results. First, the sedentary behavior patterns (e.g., prolonged uninterrupted sedentary behavior) can lead to a significant decrease in vascular endothelial function, which in turn results in an increased risk of cardiovascular disease [64]. However, according to the descriptive statistics of studies in this review, most of the included studies (23 out of 34 studies) used subjective methods to assess sedentary behavior. This measure does not accurately capture the time duration and the intermittency of sedentary behavior, making the relationship inconsistent. Second, the analytical methods also contribute to the differences. The influence of sedentary behavior on metabolic syndrome/cardiovascular disease risk factors is moderated by many confounders (e.g., gender, BMI, physical activity, sleep). The majority of articles (20 studies) adjusted for confounders such as gender, age, lifestyle (e.g., smoking, alcohol consumption, etc.). Only fourteen studies controlled the impact of physical activity and sleep. The co-linear and interdependent relationships between physical activity, sedentary behavior, and sleep make the effects of sedentary behavior on the risk factors for metabolic syndrome/cardiovascular disease quite different among studies [65]. Further research is needed to identify potential confounding factors of the relationship between sedentary behavior and metabolic syndrome/cardiovascular disease risk factors in young adults.

The limited number of studies (two studies) that have investigated the relationship between sedentary behavior and cognitive function precluded a consistent conclusion from being drawn. Furthermore, the relationship between sedentary behaviors and prospective changes in cognitive function may be influenced by the type of sedentary behavior. Previous longitudinal study has shown that when adults operate the mouse or locate icons on a computer screen, they can exercise basic psychomotor and sensory skills; when they figure out how to utilize computer processes to perform tasks, their learning, memory, and executive function can be improved [66] Hence, through the activation of learning, memory, and psychomotor processes, the sedentary behavior of computer use may serve as a form of mental stimulation that can train and maintain cognitive abilities [67] while during the other type of sedentary behavior such as watching TV, the rapid change images and sounds on television cause the brain to be more alert but less focused, which decreases the working memory of adults and leading to a decline in cognitive function [68]. 

In addition, the insufficient evidence for the relationship between sedentary behavior and emotional disorder may also be related to the type of sedentary behavior. When spending time using smartphones and tablets, adults are in a passive mental state that could disturb normal neurocognitive development processes, which could be linked to stress and depression [69]. In contrast, the reading and writing time could promote autonomy and self-reliance, improve the sense of accomplishment and well-being, and finally, reduce mood disorders [69]. Nineteen out of all the included studies in the current review analyzed the effects of overall sedentary behavior on health outcomes. Therefore, we cannot draw consistent results from these studies. Furthermore, age may have confounded the association between sedentary behavior and emotional disorder. Previous systematic reviews have demonstrated that the sedentary behavior of television viewing is associated with unfavorable psychosocial health among children and adolescents [70,71]. However, for young adults, watching TV (which is likely to be used for leisure purposes) may be time for adults with depressive symptoms to relax, which is not detrimental to emotional health [27]. Future studies are required to capture the effects of different types of sedentary behaviors and age on cognitive function and emotional disorder.

There are several strengths and limitations of this review. The strengths include the systematic approach in the extensive literature search and in the assessment of the methodological quality of each study, and the use of the best evidence systems to draw conclusions. Moreover, this review only included longitudinal studies, thus providing insights into the changes over time in the impact of sedentary behavior on the health outcomes of young adults. However, this review still had some limitations. First, although a comprehensive search of the published literature was conducted, literature using other keywords may not have been included. Second, only the published literature in English was included, certain studies that could have added relevant information to this field may have been discarded.

## 5. Conclusions

We systematically collated the relevant literature on the relationship between sedentary behavior and health outcomes among young adults. The review showed moderate strength evidence of the negative relationship between sedentary behavior and physical fitness. In the future, more research is needed to objectively measure the pattern (e.g., prolonged sitting, intermittent sedentary) and type (e.g., watching TV, using smartphones and tablets) of sedentary behavior in combination with robust and standardized measures of health indicators to gain an explicit understanding of the impact of sedentary behavior on health indicators. Moreover, evidence from experimental studies and intervention trials will help inform policy decisions and provide guidelines of the dose–response between sedentary behavior and health indicators.

## Figures and Tables

**Table 1:** The study characteristics.

Study (Year)	Country	Sample	Follow-Up Duration	Type of Sedentary Behavior (and Measure)	Type of Health Outcome (and Measure)	Variables Controlled in Analysis	Statistical Analysis	Outcomes
Ki et al., 2011 [28]	United Kingdom	*n* = 7824, aged 23 years, male 50.2%, female 49.8%	22 years	TV viewing (EPAQ-2)	HDL-cholesterol, and triglycerides (non-fasting venous blood samples)	Smoking, alcohol drink, diet, sedentary behavior, activity, BMI, long-term limiting illness, birth weight	Linear regression	TV viewing and HDL cholesterol: β = −0.02, *p* < 0.001 TV viewing and triglycerides: β = 0.026, *p* < 0.01
Thomée et al., 2012 [11]	Sweden	*n* = 4163, aged 20–24 years, male 35%, female 65%	1 year	Computer use (self-report questionnaires)	Stress and symptoms of depression (Prime-MD)	Status, educational level, occupation	Cox regression	Computer use and stress: PR = 1.7 (95%CI, 1.23, 2.25) Computer use and symptoms of depression: PR = 1.9 (95%CI, 1.17, 3.03)
Van de Laar et al., 2014 [29]	The Nether lands	*n* = 373, aged 32 years, male 47.5%, female 52.5%	4 years	Television time (self-report questionnaires)	Cardiovascular risk factors (ultrasound scanner)	Gender, body height, alcohol consumption and smoking behavior, daily energy intake, physical activity	Generalized estimating equations	Television time and cardiovascular risk factors: triglycerides: β = 0.078 (0.008, 0.148), *p* *<* 0.05 Total-to-HDL cholesterol ratio: β = 0.066 (0.007, 0.125), *p* *<* 0.05 mean arterial pressure: β = 0.078 (0.007, 0.148), *p* *<* 0.05
Lyden et al., 2015 [30]	USA	*n* = 10, mean age 25.2 ± 5.7 years, male 40%, female 60%	7 days	Sitting (accelerometers)	Markers of cardiometabolic risk (oral glucose tolerance test)	MVPA	Linear regression	Sitting and plasma insulin: β = 0.91, *p* *<* 0.01 Sitting and glucose: β = 0.39, *p* = 0.16
Pouliou et al., 2012 [31]	United Kingdom	*n* = 9927, aged 23 years, male 46.7%, female 53.3%	22 years	TV viewing (EPAQ)	Blood pressure (digital oscillometer sphygmomanometer)	Birth-weight, smoking, alcohol, diet, social class, and pre-existing medical condition	Logistic regression	TV viewing and blood pressure in men: OR = 1.06 (1.00, 1.12) TV viewing and blood pressure in women: OR = 1.01 (0.94, 1.09)
Altenburg et al., 2016 [32]	The Nether lands	*n* = 7, aged 18–23 years, male	6 days	Sitting time (accelerometers)	Glucose, C-peptide, and triglycerides (blood sample)	LPA time	Generalized estimating equations	Sitting time and metabolic risk factors [β (90%CI)]: C-peptide: 0.11(0.002, 0.22), *p* *<* 0.01 glucose and triglycerides were not significantly different
Drenowatz et al., 2016 [33]	USA	*n* = 332, mean age 23.7 ± 3.7 years, male 50%, female 50%	1 year	Sedentary time (accelerometers)	BMI (kg/m^2^) Body fat percentage (fat mass/body weight)	Age, baseline sedentary time, baseline body composition, MVPA	Linear regression	Sedentary time and BMI [β]: −0.074, *p* *>* 0.05 Sedentary time and body fat percentage [β]: 0.062, *p* *<* 0.05
Hoang et al., 2016 [10]	USA	*n* = 3247, mean age 25.1 ± 3.6 years, male 43.5%, female 56.5%	25 years	Television viewing (asked)	Cognitive function (DSST, Stroop test, RAVLT)	Age, race, sex, educational level, smoking, BMI, and hypertension	Logistic regression	Television viewing and cognitive function DSST: OR = 1.64 (1.21, 2.33) Stroop: OR = 1.56 (1.13, 2.14) RAVLT: OR = 1.14 (0.86, 1.53)
DeMello et al., 2018 [34]	USA	*n* = 430, mean age 27.66 ± 3.78 years, male 49.5%, female 50.5%	1 year	Sedentary behavior (accelerometers)	Mood (profile of mood state)	Perceived stress, age, BMI and gender, employment, PA, and total stressful life events	Cross-lagged, autoregressive clustered model	Sedentary behavior and worse mood: β = 0.20, *p* = 0.01
Cleland et al., 2018 [35]	Australia	*n* = 1068, mean age 31.5 years, male 35.95%, female 64.05%	5 years	TV viewing (Self-report questionnaires)	BMI (kg/m^2^)	Gender, age, highest level of education, marital status, employment status, occupation, number of children, and current smoking	Linear regression	TV viewing and BMI [β (95%CI)]: >1 h increase (hours/week): 0.41 (0.03, 0.78)
Staiano et al., 2018 [12]	United Kingdom	*n* = 71, mean age 26.9 ± 4.5 years, male 44%, female 56%	2 years	Sedentary time (accelerometers)	Adiposity (whole-body DXA)	Age, gender, energy intake	Linear regression	Sedentary behavior was not significantly associated with any adiposity indicators (*p* > 0.05)
Whitaker et al., 2019 [36]	USA	*n* = 1922, aged 18–30 years, male 41.6%, female 58.4%	10 years	Sedentary time (accelerometers)	Cardiometabolic risk factors (fasting venous blood samples)	Sex, race, age, education, employment status, health insurance, self-reported medication uses for blood pressure, cholesterol, or diabetes mellitus, smoking status, alcohol consumption, BMI	Linear correlations	Sedentary time and Cardiometabolic risk factors [R]: 0.070, *p* *<* 0.05
Silva et al., 2019 [37]	Brazil	*n* = 3206, mean age 30.2 years, male 49.6%, female 50.4%	13 years	Sedentary time (IPAQ-L)	Waist circumference (Bod POD Scale)	Sex, family income at birth, maternal schooling at birth, maternal skin color, birth weight, socioeconomic status, achieved schooling, smoking and daily energy intake	Linear regression	Sedentary time and waist circumference: [β (95%CI)]: 1.05 (0.16, 0.012) *p* *<* 0.001
Stamatakis et al., 2012 [38]	United Kingdom	*n* = 5972, aged 23 years, male 49.3%, female 50.7%	11 years	TV viewing (interview)	cardiometabolic risk factor (non-fasting blood samples)	Sex, smoking, alcohol, CVD medication social class, MVPA and TV viewing times	Linear regression	TV viewing and cardiometabolic risk: β = 0.048 (−0.012, 0.107), *p* = 0.071
Pavey et al., 2019 [39]	Australia	*n* = 6205, mean age 24.6 ± 1.5 years, female	12 years	Sitting time (asked)	Depression (CESD-10)	Area of residence, education, marital status, number of children, smoking status, alcohol status, BMI, chronic conditions	Generalized estimating equation	Sitting time and depression [OR (95%CI)]: Sitting time ≥10 h/day: 1.41 (1.12–1.77)
Ellingson et al., 2018 [40]	USA	*n* = 271 mean age 27.8 ± 3.7 years, male 51%, female 49%	1 year	Total time spent ≤1.5 METs while awake (Accelerometers)	Mood (profile of mood states) Stress (Perceived Stress Scale)	Age, sex, race, education	Linear regression	Sedentary behavior and total mood disorder: β = 0.23, *p* = 0.001 Sedentary behavior and stress: β = 0.20, *p* = 0.006
Carter et al., 2020 [13]	USA	*n* = 75, mean age 33.6 ± 10.4 years male 44%, female 56%	1 year	Sedentary behavior (accelerometers)	Mood (PNANS, Bond–Lader); Cognitive (Stroop, ANT, N-Back Tasks)	Age, sex	Linear regression	Sedentary behavior was not significantly associated with cognitive function and mood (*p* *>* 0.05)
Uddin et al., 2020 [41]	Australia	*n* = 395, mean age 20.7 ± 1.35 years, male 55%, female 45%	1 year	Sedentary behavior (GPAQ)	Psychological distress (Kessler Psychological Distress)	Age, gender, marital status, BMI, education, occupation, income, television (TV) in bedroom, perceived health, sleep difficulties, smoking, diet	Generalized Estimating Equations	Sedentary behavior was not significantly associated with psychological distress (*p* = 0.638)
Fujii et al., 2020 [42]	Japan	*n* = 10,212, mean age 34 years, male 48.8%, female 51.2%	4.8 years	Sedentary behavior (asked)	Proteinuria (dipstick test)	Age, sex, smoking status, sleep duration, BMI, systolic blood pressure, sedentary workers, television viewing, exercise, cardiovascular diseases	Cox regression	Sedentary behavior and proteinuria: HR = 1.35, (1.11–1.63)
Vaara et al., 2020 [9]	Finland	*n* = 415, mean age 26 ± 7 years, male	7 days	≤1.5 METs (accelerometers)	Body fat content (bioelectrical impedance method) Physical fitness (indirect graded cycle ergometer test and muscular fitness tests)	Age and smoking	Linear regression	Sedentary behavior and cardiorespiratory fitness and muscular fitness: β = −0.245 (−0.338; −0.152), β = −0.193 (−0.287; −0.099) Sedentary behavior and body fat content: β = 0.42, *p* < 0.001)
Vieira et al., 2020 [43]	Brazil	*n* = 34, mean age 31.85 years, female	2 years	Sitting/lying time (accelerometers)	Body weight (electronic scale) WHR (waist/hip) blood cardiovascular (fasting blood sample)	Age, schooling, number of children, marital status, tobacco status, alcohol user, unemployment, per capita income	Multivariable mixed models	Sitting/lying time was associated with an increase in WHR, but not in body weight or blood cardiovascular risk factors.
Mars et al., 2020 [44]	United Kingdom	*n* = 1431, mean age 18.2 ± 0.5 years, male 62.6%, female 37.4%	3 years	Internet use (asked)	Depression (sMFQ) Anxiety (GAD-7)	Earlier mental health problems	Logistic regression	Internet use and anxiety: OR = 1.00 (0.99, 1.02), *p* = 0.310
Thomée et al., 2007 [45]	Sweden	*n* = 1127, aged 18–25, male 51.9% female 48.1%	1 year	Computer/ Internet use (self-report questionnaires)	Stress, depression, anxiety (Prime-MD)	Age, sex, social position	Crude prevalence ratios	Overall computer or internet use and stress (95%CI): 1.02 (0.60,1.75); Depression: 1.02 (0.60,1.75); anxiety: 0.62 (0.36, 1.05);
Endrighi et al., 2016 [46]	United Kingdom	*n* = 43, aged 18–35 years, male 55.8%, female 44.2%	4 weeks	Sedentary time (accelerometers)	Psychological distress (GHQ-28) Mood (POMS-SF)	MVPA	Linear regression	No significant associations emerged between GHQ scores and changes in sedentary time (β = 0.08, *p* = 0.62) Sedentary time was significantly associated with the POMS negative mood score (β = 0.32, *p* = 0.03)
Jeffery et al., 1998 [47]	USA	*n* = 1059, mean aged 34 years, male 18.7%, female, 81.3%	1 year	Television viewing (self-report questionnaires)	BMI (kg/m^2^)	Age, education, baseline smoking, baseline body mass index, energy intake, physical activity.	Linear regression	Significant positive relationship between hours of TV viewing and change in body mass index in high-income women (β = 0.30; 0.02, 0.58)
Ball et al., 2003 [48]	Australia	*n* = 8726, aged 18–23, women	4 years	Sitting time (self-report questionnaires)	Body weight (BMI)	Occupation, student status, marital status, parity and new mothers	ANOVA	Compared with the ‘low sitting’ group, the women who reported moderate or high sitting time were less likely to be in the weight maintainers.
Hancox et al., 2004 [49]	New Zealand	*n* = 980, birth cohort. boy 48%, girl 52%	26 years	Television viewing (self-report questionnaires)	Cardiometabolic risk (fasting blood samples)	Sex, bodyweight, physical activity	Linear regression	TV viewing time is a significant predictor of elevated cholesterol (mmol/L) at age 26 years: β (SE) = 0.09 (0.04) *p* = 0.0383 No significant association with blood pressure
Viner et al., 2005 [50]	United Kingdom	*n* = 8158, birth cohort, male, 48.7%, female 51.3%	5, 10, and 30 years	Television viewing (self-report questionnaires)	BMI (Self-report)	Gender, birth weight, social class, educational status	Linear regression	β (95% CI) on weekends = 0.04 (0.03, 0.06), *p* < 0.001; β (95% CI) on weekdays = 0.03 (0.01, 0.05), *p* = 0.001
Boone et al., 2016 [51]	USA	*n* = 9605, mean aged 21.4 years, male 50.8%, female 49.2%	6 years	Screen time (self-report questionnaires)	Obesity (BMI)	Age, race/ethnicity, household income, and highest parental education	Logistic regression	Screen time hours had a stronger influence on incident obesity in females [OR (95% CI): OR 4 vs. 40 h = 0.58 (0.43, 0.80)] than males [OR (95% CI): OR 4 vs. 40 h = 0.78 (0.61, 0.99)]
Beunza et al., 2007 [52]	Spain	*n* = 6742, Mean age 33.3 years, male 38.2%, female 61.8%	Mean 40 months	TV viewing, computer use, driving, sleeping (self-report questionnaires)	Hypertension (Follow-up questionnaires)	Age, gender, BMI, physical activity, family history of hypertension, hypercholesterolemia, smoking status, intake of sodium alcohol, low-fat dairy, fruit, vegetable, and olive oil	Cox regression	HR (95% CI) for incident hypertension and total sedentary behavior <14.2 h/day = 1.00 (ref) >21 h/day = 1.48 (1.01, 2.18)
Landhuis et al., 2008 [53]	New Zealand	*n* = 1037, Born April 1972–March 1973, male 51.6%, female 48.4%	27 years	Television viewing (asked)	Fitness (cycle ergometer) obesity (BMI ≥ 30 kg/m^2^)	Childhood socioeconomic status, early BMI, and parental BMI	Logistic regression	Childhood viewing predicted both adult obesity (OR 95%CI = 1.30; 1.07, 1.58) and adult poor fitness (OR 95%CI = 1.41; 1.17, 1.69).
Parsons et al., 2008 [54]	United Kingdom	*n* = 11,301, birth cohort, male 50.7%, female 49.3%	16, 23, and 33 years	Television viewing (self-report questionnaires)	BMI and central adiposity (Calculated and indexed by waist and hip circumferences)	Maternal BMI, social class, puberty status, physical activity, alcohol consumption, smoking status, healthy eating score	Linear regression	TV viewing at 23 years was significantly associated with waist–hip ratio at age 45 years: ≥5 times a week = 0.006 (men), 0.004 (women)
Crawford et al., 1999 [55]	Australia	*n* = 881, mean aged 34.3 years, male 20%, female 80%	3 years	Television viewing (self-report questionnaires)	BMI (kg/m^2^)	Dietary, age, education, smoking, baseline BMI	Linear regression	There were no significant relationships between change in BMI and TV viewing
Primack et al., 2021 [56]	USA	*n* = 990, mean age 27.0 ± 2.7 years, male 45.9%, female 55.1%	6 months	Social media use (asked)	Depression (Questionnaire)	Age, sex, race and ethnicity, educational level, household income, relationship status, living situation, and adverse childhood experiences	Logistic regression	Social media use and depression: OR = 1.04 (0.78, 1.38)

**Table 2:** The overall scores of the methodological quality assessment for the included studies.

Reference	Source Population	Recruitment	Participation Rate	Description Baseline Sample	Numbers at Follow-Up	Follow-up Duration	Response rate at Follow-Up	Not-Selective non-Response	Measure SB	SB Measured before Health Outcome	Measure Health Outcome	Appropriate Statistical Model	Cases at Least 10 Times	Point Estimates and Measures of Variability	No Selective Reporting of Results	Total	Percentage ‘+’
Carter et al., 2020 [13]	+	?	+	+	+	+	+	−	+	+	−	+	−	+	+	11	73
Whitaker et al., 2019 [36]	+	+	–	+	+	+	−	−	+	+	?	+	+	+	+	11	73
Ki et al., 2011 [28]	+	?	+	+	?	+	+	−	−	+	+	+	+	+	−	10	67
Stamatakis et al., 2012 [38]	+	?	+	+	?	+	−	+	−	+	+	+	+	+	−	10	67
Drenowatz et al., 2016 [33]	+	?	–	+	+	+	−	+	+	+	?	+	+	+	−	10	67
Uddin et al., 2020 [41]	+	?	+	+	+	+	−	+	−	+	−	+	+	+	−	10	67
Beunza et al., 2007 [52]	+	+	–	+	−	+	+	−	−	+	−	+	+	+	+	10	67
Silva et al., 2019 [37]	+	?	+	?	+	+	−	−	−	+	−	+	+	+	+	9	60
Pouliou et al., 2012 [31]	+	?	+	+	?	+	−	−	−	+	+	+	+	+	−	9	60
Cleland et al., 2018 [35]	+	?	–	+	+	+	−	+	−	+	−	+	−	+	+	9	60
Vaara et al., 2020 [9]	+	?	+	+	?	+	−	−	+	+	?	+	+	+	−	9	60
Ellingson et al., 2018 [40]	+	?	–	+	−	+	−	−	+	+	−	+	+	+	+	9	60
Altenburg et al., 2016 [32]	+	?	–	+	+	+	−	−	+	+	?	+	−	+	+	9	60
Fujii et al., 2021 [42]	–	?	–	+	?	+	+	−	−	+	?	+	+	+	+	8	53
Mars et al., 2020 [44]	+	?	+	?	+	+	−	−	−	+	−	+	+	+	−	8	53
Hoang et al., 2016 [10]	+	?	–	+	−	+	−	+	−	−	+	+	+	+	−	8	53
Thomée et al., 2012 [45]	+	+	–	?	+	+	−	−	−	+	−	+	+	+	−	8	53
DeMello et al., 2018 [34]	+	?	–	+	+	+	−	−	+	−	−	+	+	+	−	8	53
Pavey et al., 2019 [39]	+	?	–	+	−	+	−	−	−	+	−	+	+	−	+	7	47
Primack et al., 2021 [56]	–	?	+	+	?	+	−	−	−	+	−	+	+	+	−	7	47
Staiano et al., 2018 [12]	–	?	–	+	−	+	−	−	+	+	−	+	−	+	+	7	47
Van de Laar et al., 2014 [29]	+	?	–	+	?	+	−	−	−	+	−	+	+	+	−	7	47
Lyden et al., 2015 [30]	–	–	–	?	−	+	+	−	+	+	?	+	−	+	+	7	47
Hancox et al., 2004 [49]	+	?	+	−	+	+	+	?	−	−	?	+	−	+	−	7	47
Endrighi et al., 2016 [46]	+	-	–	?	−	+	−	−	+	+	−	+	?	+	−	6	40
Parsons et al., 2008 [54]	+	-	+	?	?	+	?	−	−	+	−	+	−	+	−	6	40
Landhuis et al., 2008 [53]	+	-	+	−	−	+	+	−	−	−	−	+	−	+	−	6	40
Thomée et al., 2007 [45]	+	?	–	−	+	+	−	−	−	+	−	+	−	+	−	6	40
Vieira et al., 2020 [43]	–	?	–	+	+	+	−	+	+	−	?	+	−	−	−	6	40
Boone et al., 2016 [51]	-	–	–	+	−	+	+	−	−	+	−	+	−	+	−	6	40
Ball et al., 2003 [48]	+	?	–	+	−	+	−	−	−	+	−	−	−	+	−	5	33
Viner et al., 2005 [50]	+	?	–	?	+	+	−	−	−	−	−	+	−	+	−	5	33
Jeffery et al., 1998 [47]	–	?	–	+	−	+	−	−	−	−	−	+	−	+	−	4	27
Crawford et al., 1999 [55]	–	?	–	?	−	+	−	−	−	−	−	+	−	+	−	3	20

“+” positive, thoroughly and clearly described, “−“ negative, not illustrated, “?” insufficiently described.

**Table 3:** The results of the data synthesis.

Health Indicator	Association	Studies (Primary Author, Publication Year)	Association (%)	Evidence
Adiposity indicators	+	Vaara et al., 2020 [9] ^a^; Jeffery et al., 1998 [47] ^b^; Viner et al., 2005 [50] ^b^; Parsons et al., 2008 [54] ^b^; Ball et al., 2003 [48] ^b^; Boone et al., 2016 [51] ^b^; Landhuis et al., 2008 [53] ^b^	7/12 58.3%	Insufficient
	0	Silva et al., 2019 [37] ^a^; Drenowatz et al., 2016 [33] ^a^; Cleland et al., 2018 [35] ^a^; Staiano et al., 2018 [12] ^b^; Crawford et al., 1999 [55] ^b^	5/12 41.7%	
Physical fitness	−	Vaara et al., 2020 [9] ^a^; Landhuis et al., 2008 [53] ^b^	2/2 100%	Moderate
Metabolic syndrome/ cardiovascular disease risk factors	+	Whitaker et al., 2019 [36] ^a^; Fujii et al., 2021 [42] ^a^; van de Laar et al., 2014 [29] ^b^; Ki et al., 2011 [28] ^a^; Hancox et al., 2004 [49] ^b^	5/11 45.4%	Insufficient
	0	Pouliou et al., 2012 [31] ^a^; Beunza et al., 2007 [52] ^a^; Altenburg et al., 2016 [32] ^a^; Stamatakis et al., 2012 [38] ^a^; Lyden et al., 2015 [30] ^b^; Vieira et al., 2020 [43] ^b^	6/11 54.6%	
Cognitive function	−	Hoang et al., 2016 [10] ^a^	1/2 50%	Insufficient
	0	Carter et al., 2020 [13] ^a^	1/2 50%	
Emotional disorder	+	Ellingson et al., 2018 [40] ^a^; DeMello et al., 2018 [34] ^a^; Pavey et al., 2019 [39] ^b^; Primack et al., 2021 [56] ^b^	4/10 40%	Insufficient
	0	Carter et al., 2020 [13] ^a^; Uddin et al., 2020 [41] ^a^; Mars et al., 2020 [44] ^a^; Thomée et al., 2012 [11] ^a^; Endrighi et al., 2016 [46] ^b^, Thomée et al., 2007 [45] ^b^	6/10 60%	

“+” positive association; “−” negative association; “0” no association; ^a^ high quality; ^b^ low quality.

## Data Availability

The raw data supporting the conclusions of this article are available from the corresponding author on reasonable request.

## Supplementary Materials

The following supporting information can be downloaded at: https://www.mdpi.com/article/10.3390/healthcare10081480/s1, Table S1: Search strategies.
