# Supplementary material for: Sedentary Behaviors and Health Outcomes among Young Adults: A Systematic Review of Longitudinal Studies
Source: Healthcare (Basel). 2022 Aug 6;10(8):1480. doi: 10.3390/healthcare10081480 (PMC9408295; doi:10.3390/healthcare10081480)
Supplement: Supplementary file 1 [file healthcare-10-01480-s001.zip › healthcare-1836401-supplementary.pdf]

healthcare

MDPI

Table S1: Search strategies.

sedentary behavior: "physical inactivity" OR sedenta* OR "sedentary behaviour" OR "sedentary lifestyle*" OR sitting OR television OR "screen time" OR video game OR social media OR internet<br>health outcomes: "health* outcome" OR BMI OR obesity* OR "physical fitness" OR "physical function" OR "muscle strength" OR "muscle endurance" OR cardiorespiratory* OR metabolic* OR hypertension OR "diabete*" OR "cardiovascular disease*" OR "health-related" OR disease* OR "mental health*" OR depression* OR anxiety* OR stress OR "self-esteem" OR "self-efficacy" OR "cognitive function*"<br>young adult: "young adult*" OR adult* OR "middle aged" OR "college student" OR postgraduate*
**Web of Science: 3385**
((TI=("physical inactivity" OR sedenta* OR "sedentary behaviour" OR "sedentary lifestyle*" OR sitting OR television OR "screen time" OR "video game" OR "social media" OR internet)) AND AB=("health* outcome" OR BMI OR obesity* OR "physical fitness" OR "physical function" OR "muscle strength" OR "muscle endurance" OR cardiorespiratory* OR metabolic* OR hypertension OR "diabete*" OR "cardiovascular disease*" OR "health-related" OR disease* OR "mental health*" OR depression* OR anxiety* OR stress OR "self-esteem" OR "self-efficacy" OR "cognitive function*")) AND AB=(young adult* OR adult* OR "middle aged" OR "college student" OR postgraduate*)
**APA PsycInfo: 1055**
TI="physical inactivity" OR sedenta* OR "sedentary behaviour" OR "sedentary lifestyle*" OR sitting OR television OR "screen time" OR "video game" OR "social media" OR internet AND AB="health* outcome" OR BMI OR "waist circumference" OR "body composition" OR obesity* OR "physical fitness" OR "physical function" OR "muscle strength" OR "muscle endurance" OR cardiorespiratory* OR metabolic* OR hypertension OR "diabete*" OR "cardiovascular disease*" OR "health-related" OR disease* OR "mental health*" OR depression* OR anxiety* OR stress OR "self-esteem" OR "self-efficacy" OR "cognitive function*" AND AB="young adult"* OR adult* OR "middle aged" OR "college student" OR postgraduate*
**MEDLINE: 2706**
TI="physical inactivity" OR sedenta* OR "sedentary behaviour" OR "sedentary lifestyle*" OR sitting OR television OR "screen time" OR "video game" OR "social media" OR internet AND AB="health outcome" OR BMI OR "waist circumference" OR "body composition" OR obesity* OR "physical fitness" OR "physical function" OR "muscle strength" OR "muscle endurance" OR cardiorespiratory* OR metabolic* OR hypertension OR diabete* OR "cardiovascular disease*" OR "health-related" OR disease* OR "mental health*" OR depression* OR anxiety* OR stress OR "self-esteem" OR "self-efficacy" OR "cognitive function*" AND AB="young adult"* OR adult* OR "middle aged" OR "college student" OR postgraduate*)
**Pubmed: 3130**
(("Sedentary Behavior"[Mesh] OR sitting[Title] OR television[Title] OR "screen time"[Title) AND ("Body Mass Index"[Mesh] OR "health outcome" OR "waist circumference" OR obesity* OR "physical health*" OR "physical function" OR "muscle strength" OR "muscle endurance" OR cardiorespiratory* OR "Metabolic Syndrome"[Mesh] OR hypertension OR diabete OR "cardiovascular disease*" OR "health-related"OR disease* OR "mental health*" OR depress* OR anxiety* OR stress OR "self-esteem" OR "self-efficacy" OR "Cognition"[Mesh])) AND ("young adult*"[Title/Abstract] OR adult*[Title/Abstract] OR "middle aged"[Title/Abstract] OR "college student"[Title/Abstract] OR "postgraduate*"[Title/Abstract])
**Cochrane Library: 1589**
"physical inactivity" OR sedentar* OR "sedentary behaviour" OR "sedentary lifestyle*" OR sitting OR television OR "screen time" OR "video game" OR "social media" OR internet in Record Title AND "health* outcome" OR BMI OR "waist circumference" OR "body composition" OR obesity* OR "physical fitness" OR "physical function" OR "muscle strength" OR "muscle endurance" OR cardiorespiratory* OR metabolic* OR hypertension OR "diabete*" OR "cardiovascular disease*" OR "health-related" OR disease* OR "mental health*" OR depression* OR anxiety* OR stress OR "self-esteem" OR" self-efficacy" OR "cognitive function*" in Title Abstract Keyword AND "young adult"* OR adult* OR "middle aged " OR "college student "OR postgraduate* in Title Abstract Keyword
**Embase: 3521**
('physical inactivity':ti OR sedenta*:ti OR 'sedentary behaviour':ti OR 'sedentary lifestyle*':ti OR sitting:ti OR television:ti OR 'screen time':ti OR 'video game':ti OR 'social media':ti OR internet:ti) AND ('health* outcome':ab,ti OR

bmi:ab,ti OR 'waist circumference':ab,ti OR 'body composition':ab,ti OR obesity*:ab,ti OR 'physical fitness':ab,ti OR 'physical function':ab,ti OR 'muscle strength':ab,ti OR 'muscle endurance':ab,ti OR cardiorespiratory*:ab,ti OR metabolic*:ab,ti OR hypertension:ab,ti OR 'diabete*':ab,ti OR 'cardiovascular disease*':ab,ti OR 'health-related':ab,ti OR disease*:ab,ti OR 'mental health*':ab,ti OR depression*:ab,ti OR anxiety*:ab,ti OR stress:ab,ti OR 'self-esteem':ab,ti OR 'self-efficacy':ab,ti OR 'cognition*':ab,ti) AND ('young adult*':ab,ti OR adult*:ab,ti OR 'middle aged':ab,ti OR 'college student':ab,ti OR postgraduate*:ab,ti)
